# Environmental Conditions Modulate the Transcriptomic Response of Both *Caulobacter crescentus* Morphotypes to Cu Stress

**DOI:** 10.3390/microorganisms9061116

**Published:** 2021-05-21

**Authors:** Laurens Maertens, Pauline Cherry, Françoise Tilquin, Rob Van Houdt, Jean-Yves Matroule

**Affiliations:** 1 Microbiology Unit, Interdisciplinary Biosciences, Belgian Nuclear Research Centre (SCK CEN), 2400 Mol, Belgium; laurens.maertens@sckcen.be (L.M.); rvhoudto@sckcen.be (R.V.H.); 2 Research Unit in Microorganisms Biology (URBM), Narilis Institute, University of Namur, 5000 Namur, Belgium; pauline.cherry@unamur.be (P.C.); francoise.tilquin@unamur.be (F.T.)

**Keywords:** *Caulobacter*, copper, RNA-Seq, transcription, environment, morphotype

## Abstract

Bacteria encounter elevated copper (Cu) concentrations in multiple environments, varying from mining wastes to antimicrobial applications of copper. As the role of the environment in the bacterial response to Cu ion exposure remains elusive, we used a tagRNA-seq approach to elucidate the disparate responses of two morphotypes of *Caulobacter crescentus* NA1000 to moderate Cu stress in a complex rich (PYE) medium and a defined poor (M2G) medium. The transcriptome was more responsive in M2G, where we observed an extensive oxidative stress response and reconfiguration of the proteome, as well as the induction of metal resistance clusters. In PYE, little evidence was found for an oxidative stress response, but several transport systems were differentially expressed, and an increased need for histidine was apparent. These results show that the Cu stress response is strongly dependent on the cellular environment. In addition, induction of the extracytoplasmic function sigma factor SigF and its regulon was shared by the Cu stress responses in both media, and its central role was confirmed by the phenotypic screening of a *sigF*::Tn*5* mutant. In both media, stalked cells were more responsive to Cu stress than swarmer cells, and a stronger basal expression of several cell protection systems was noted, indicating that the swarmer cell is inherently more Cu resistant. Our approach also allowed for detecting several new transcription start sites, putatively indicating small regulatory RNAs, and additional levels of Cu-responsive regulation.

## 1. Introduction

Copper (Cu) is an essential micronutrient for all living organisms, but elevated intracellular concentrations can quickly lead to toxic effects. Thus, it is imperative to maintain a strictly controlled Cu ion homeostasis. Elevated Cu ion concentrations in the environment can originate from both natural and anthropogenic sources, with the latter including sources such as mining, smelting, domestic waste emission, and pesticide runoff [1]. Since Cu contamination in the environment is a growing issue, it is necessary to gain a thorough understanding of the biological response to this stressor. Microorganisms can also come into contact with toxic Cu ion concentrations in macrophages, which use the metal to combat invading microbes [2]. Finally, new Cu-based antimicrobial therapies are in development as alternatives for traditional organic antibiotics [3]. Overall, the influence of a variable environment on the microbial Cu stress response remains elusive.

Recent studies have asserted a large diversity of cellular targets of Cu toxicity. Giachino and Waldron [4] emphasized the impact on the cell envelope, where Cu ions interfere with peptidoglycan and lipoprotein maturation [5,6]. In addition, Cu ions can displace other metals from binding sites in proteins [7,8]. Especially solvent-exposed Fe-S clusters could be vulnerable to this aspect of toxicity [9]. Finally, Lemire et al. [10] highlighted the role of oxidative stress following the generation of reactive oxygen species (ROS) from the rapid intracellular cycling between Cu^+^ and Cu^2+^ [11,12].

Bacteria have evolved several distinct defense systems against toxic Cu ion concentrations. Nies and Silver [13] distinguished six general metal resistance strategies, of which active ion export from the cytoplasm and periplasm, sequestration, and enzymatic alteration of the toxic ion are most prevalent. Indeed, these strategies have all been found to play their roles in bacterial Cu ion homeostasis, though not necessarily all in the same species. Cu^+^ export from the cytoplasm is achieved by P-type ATPases such as CopA [14]. In addition, heavy metal efflux-resistance nodulation cell division (HME-RND) systems such as CusCBA and SilCBA have been described to play a role in removing intracellular Cu^+^ ions [15,16,17]. Chaperones such as CopZ are involved in intracellular Cu^+^ sequestration, while multi-copper oxidases such as CueO oxidize Cu^+^ to the less toxic Cu^2+^ ion [18,19,20]. An overview of bacterial Cu ion resistance mechanisms can be found in Gillet et al. (2019) [21].

*Caulobacter* spp. have been isolated from highly diverse environments, such as metal-contaminated sites, including deep subsurface sediments and a gold mine [22,23,24]. In the lab strain *Caulobacter crescentus* NA1000, metal resistance mechanisms against Cd^2+^, Cr^6+^, U^6+^ and Cu^2+^ have been detected [25]. In the lab, we showed that *C. crescentus* NA1000 employs a bimodal response to Cu stress, where the stalked cell type relies strongly on the PcoAB system for detoxification, while the swarmer cell type favors a negative chemotaxis to escape toxic Cu concentrations [26].

Prior studies have highlighted important features of the Cu stress response in *C. crescentus* NA1000, but a genome-wide response has not yet been examined. In addition, there is currently a lack of knowledge about the role of environmental conditions such as culture medium on the Cu stress response. This is especially relevant since *Caulobacter* species have been isolated from diverse environments, often with dissimilar chemical makeup. Here, we performed tagRNA-seq, which allows for differential gene expression analysis as well as the accurate delineation of transcription start sites, of synchronized swarmer (SW) and stalked (ST) NA1000 cells stressed by sub-lethal Cu concentrations in a mineral medium (M2G) and a complex medium (PYE).

## 2. Materials and Methods

### 2.1. Bacterial Strains and Growth Conditions

*Caulobacter crescentus* NA1000 and a *sigF*::Tn*5* mutant (lab collection) were routinely cultured in either PYE medium or M2G medium at 30 °C on an orbital shaker at 180 rpm [27]. Growth in the presence of Cu was evaluated by diluting exponential growth phase cultures to a final optical density of 0.05 at 660 nm (OD_660_) in either PYE or M2G medium containing different CuSO_4_.5H_2_O concentrations and recording OD_660_ every 10 min for 24 h at 30 °C under continuous shaking in a Epoch 2 absorbance reader (BioTek Instruments Inc., Winooski, VT, USA).

### 2.2. Synchronization and RNA Extraction

**Figure 1:**
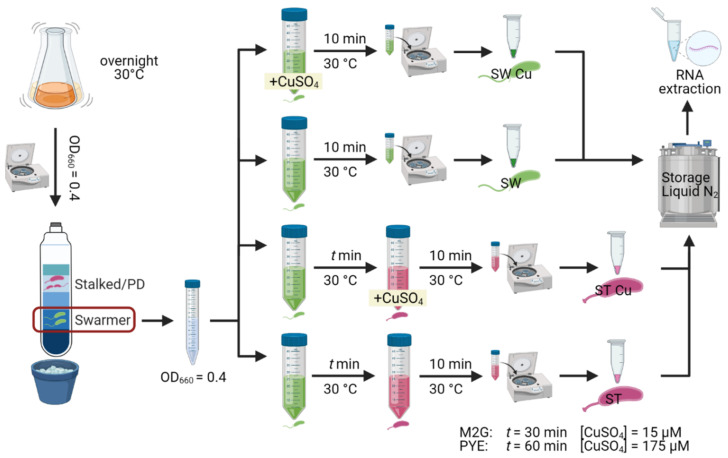
Overview of tagRNA-seq experimental setup.

For experiments with PYE medium, NA1000 overnight cultures in PYE medium were diluted in fresh PYE medium to an OD_660_ of 0.1 and allowed to grow to an OD_660_ of 0.4. Bacterial cultures were synchronized in order to isolate the swarmer fraction of cells (Evinger & Agabian, 1977), which was verified by light microscopy. The isolated swarmer cell suspension was diluted to an OD_660_ of 0.4 with PYE medium, and divided into four equal volumes. To one volume, a CuSO_4_ solution was added to a concentration of 175 µM, while to a second volume, an equivalent amount of H_2_O was added. After 10 min at 30 °C, these suspensions were centrifuged for 3 min at 8000 g and the resulting cell pellets were flash frozen. The two remaining volumes were allowed to grow at 30 °C for 40 min to enable differentiation to the stalked cell type, which was verified by light microscopy. Then, to one volume a CuSO_4_ solution was added to a concentration of 175 µM, while an equivalent amount of H_2_O was added to the other volume. After 10 min at 30 °C, these stalked cell suspensions were pelleted and flash frozen as described above (Figure 1). For experiments with M2G, an identical protocol was followed, but CuSO_4_ concentrations were decreased to 15 µM and swarmer cell differentiation time was increased to one hour. These adaptations were required to achieve similar phenotypic effects in both media (Figure 1).

The resulting cell pellets were flash frozen until resuspension in 40 µL of a 20 mg/mL proteinase K solution (Avantor^®^, Radnor, PA, USA) with 1 µL of undiluted Ready-Lyse Lysozyme solution (Lucigen^®^, Middlesex, UK), and lysis was allowed to proceed for 10 min in a shaking incubator at 37 °C and 600 rpm. Total RNA was retrieved from the cell suspensions using the *mir*Vana miRNA Isolation kit (Invitrogen™, Carlsbad, CA, USA). RNA integrity was verified by running the samples on an Agilent 2100 Bioanalyzer Nano chip (Agilent, Machelen, Belgium), and only samples with RIN values above 9 were accepted for sequencing.

### 2.3. Differential RNA Sequencing and Read Mapping

A modified tagRNA-seq protocol was performed by Vertis Biotechnologie AG, Germany, as described in [28]. Because of the different sequencing adapters in combination with RNA fragmentation, 3 read libraries were acquired for every sample. One library contained all reads originating from the 5′ end of 5′ monophosphate group RNA molecules (processing start sites, PSS reads), a second library contained all reads originating from the 5′ end of 5′ triphosphate group RNA molecules (transcription start sites, TSS reads), and a third library contained all reads from non-tagged RNA (mostly resulting from RNA fragmentation after differential adapter tagging). Trimmomatic version 0.36 [29] was used to remove adapter sequences from all three read libraries, resulting in unpaired reads of 55–75 nt. Read quality was evaluated with FastQC (version 0.10.0; https://www.bioinformatics.babraham.ac.uk/projects/fastqc/, accessed on 1 April 2020). Trimmed reads were then aligned to the *C. crescentus* NA1000 reference genome (NCBI accession number CP001340) with bwa version 0.7.12 [30], using default parameters. Reads mapping to multiple loci were removed from their respective libraries. The RNA-seq datasets generated and analyzed for this study are available from the NCBI Sequence Read Archive (SRA) under accession number PRJNA721587.

### 2.4. Differential Gene Expression Analysis

For every sample, reads from PSS, TSS, and unassigned libraries were compiled into one composite library. Read coverage counts were calculated with the featureCounts function of the Rsubread package for R [31], and subsequently normalized using the TMM method [32]. Options—isStrandSpecific was set to TRUE, and otherwise standard options were used. Differential expression was calculated using edgeR and limma [33,34,35], with the *treat* method used in addition (lfc = 1) [36]. Genes were found to be differentially expressed if they showed an FDR value lower than 0.05 and a log_2_ fold change (logFC) value either higher than 1 or lower than −1. TSSs were detected using a Python script, as previously described [28]. Principal component analysis was performed using the prcomp function in R, with options ‘scale’ and ‘center’ set to TRUE, and with the normalized read counts from featureCounts as input.

### 2.5. Reverse Trancription Quantitative Real-Time PCR

RNA (2 µg) isolated from synchronized *C. crescentus* was incubated with DNAse I (Thermo Scientific, Merelbeke, Belgium) for 30 min at 37 °C. DNAse I was then inactivated with 50 mM EDTA for 10 min at 65 °C. Subsequently, RNA was subjected to reverse transcription using MultiScribe Reverse Transcriptase (Applied Biosystems^®^, Foster City, CA, USA) with random primers (as described by the manufacturer). A total of 300 ng of cDNA was mixed with Takyon No Rox SYBR MasterMix dTTP Blue (Eurogentec, Seraing, Belgium) and the appropriate primer sets (Supplementary Table S4) and used for qPCR in a LightCycler96 (Roche, Basel, Switzerland). Forty-five PCR cycles were performed (95 °C for 10 s, 60 °C for 10 s and 72 °C for 10 s). Primer specificity was checked by melting curves analysis. Relative gene expression levels between different samples were calculated with the 2^−^^ΔΔ^^Ct^ method, using the *mreB* gene as a reference. Three technical replicates were analysed for each sample.

### 2.6. Data Visualization

**Figure 2:**
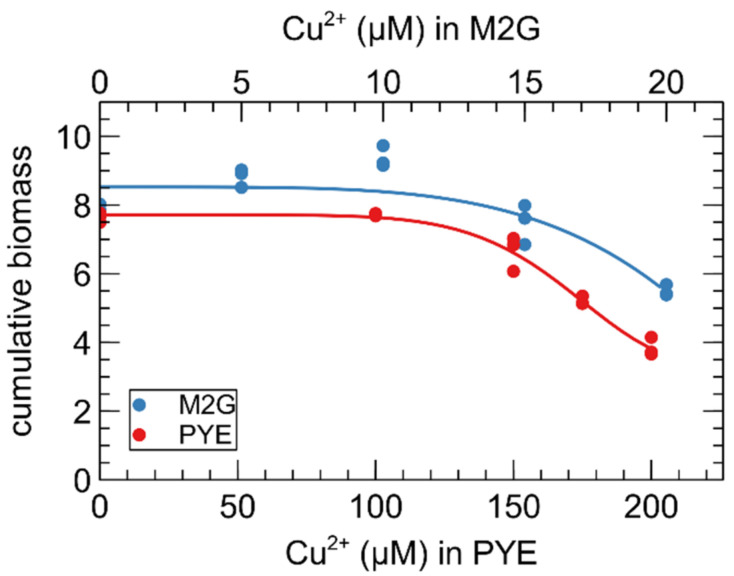
Effect of CuSO_4_ concentration on the growth of *C. crescentus* in PYE (red) and M2G (blue) medium. Cumulative biomass (time × OD_660_) is the empirical area under the growth curve (0–24 h) for three biological replicates. A four-parameter Weibull distribution was used for non-linear regression.

**Figure 3:**
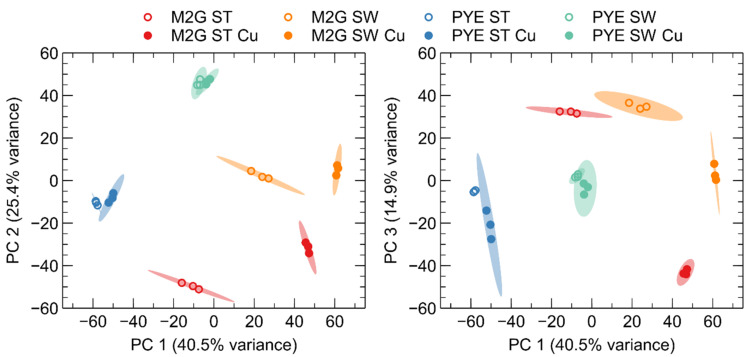
Principal component analysis (PCA) of the log2-transformed normalized gene expression values between the different control (open symbols) and Cu-treated (closed symbols) *C. crescentus* samples in M2G and PYE medium. PCA plots show the variance of the three biological replicates with 95% confidence level (colored ellipses). The percentages on each axis represent the percentages of variation explained by the principal components.

**Figure 4:**
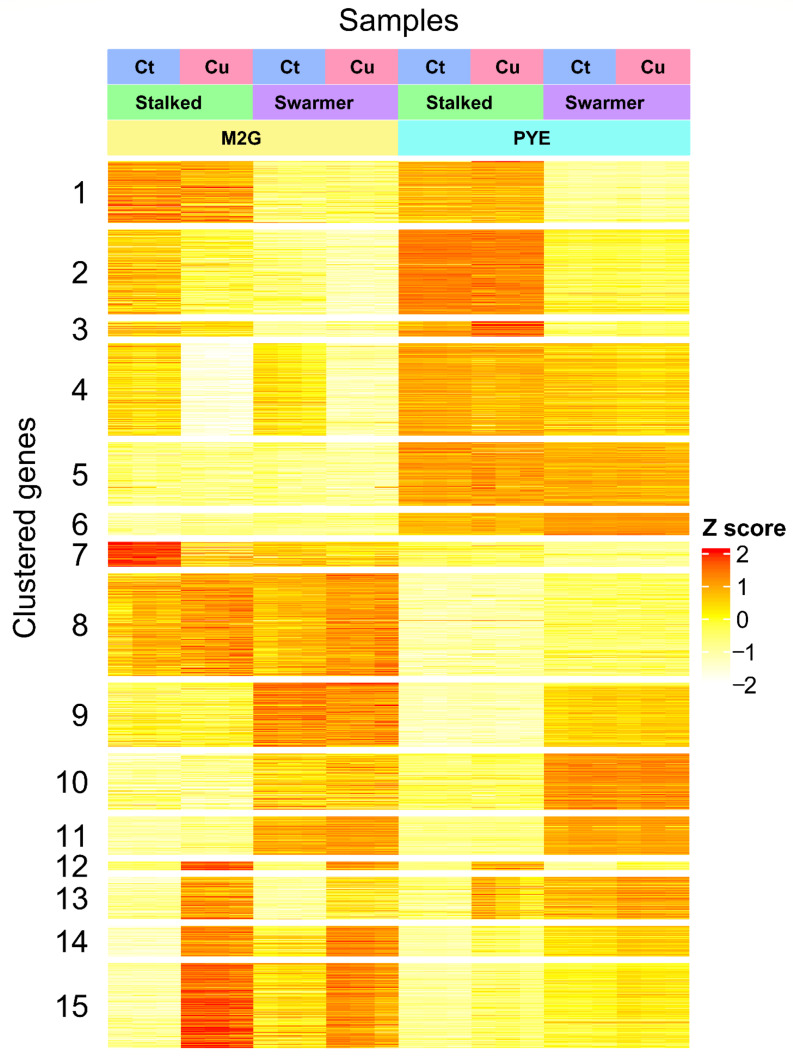
Read coverage heatmap of the 2000 most variable genes in 15 clusters. Z scores were calculated by subtracting row-average value from corresponding cell value and dividing by row standard deviation. Fifteen gene clusters were created by K-means clustering. Experimental conditions: Cu stress (blue), no Cu stress (red); cell type: stalked cells (green), swarmer cells (purple); medium: M2G medium (yellow), PYE medium (teal).

**Figure 5:**
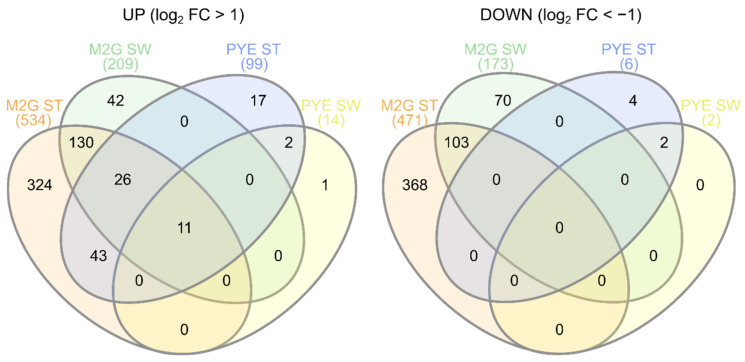
Venn diagrams showing overlap between upregulated (**left**) and downregulated (**right**) genes upon Cu stress in all experimental conditions.

**Figure 6:**
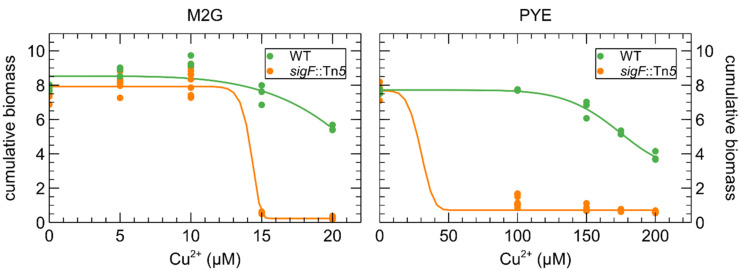
Effect of CuSO_4_ concentration on the growth of *C. crescentus* (green) and a *sigF*::Tn5 mutant (orange) in M2G (**left**) and PYE (**right**) medium. Cumulative biomass (time × OD_660_) is the empirical area under the growth curve (0–24 h) for three biological replicates. A four-parameter Weibull distribution was used for non-linear regression.

**Figure 7:**
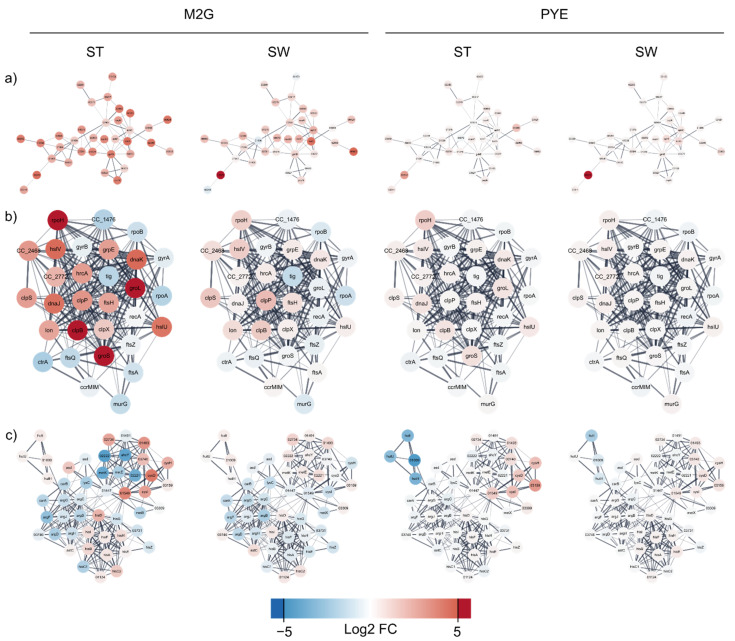
Analysis of interacting gene networks related to oxidative stress (**a**), protein misfolding, proteome rearrangement and chaperones (**b**), and the amino acid metabolism (**c**) of the *C. crescentus* differentially expressed gene dataset. Color coding is based on log2 fold change in Cu stress.

Figure 1 was created with biorender.com. Figure 2 was created with Veusz 3.3.1 based on data generated with the growthcurver package [37]. Figure 3 was created with Veusz 3.3.1 based on data generated with the prcomp function for the R project for statistical computing and the car package [38]. Figure 4 was created with the ComplexHeatmap package [39], using the internal k-means clustering method at row level. Figure 5 was generated with Inkscape v1.0.1. Figure 6 was created with Veusz 3.3.1 based on data generated with the growthcurver package [37]. Figure 7 was constructed with the STRING database output [40] that was color coded using Cytoscape 3.8.2 and combined using Inkscape 1.0.1.

## 3. Results and Discussion

### 3.1. Growth of C. crescentus in the Presence of Cu

The effect of Cu on the growth of *C. crescentus* in M2G and PYE medium was compared via biomass accumulation after 24 h (Figure 2). It is immediately clear that more CuSO_4_ must be supplemented to the complex PYE medium to achieve similar deleterious effects. Interestingly, there seems to be a hormesis effect of CuSO_4_ supplementation in M2G medium for 5 and 10 µM of Cu (*p*-value = 0.034 and 0.002, respectively). The Cu concentration for the subsequent transcriptome study were chosen to elicit a “moderate stress” in both media.

### 3.2. Read Coverage Analysis

In order to study the effects of growth conditions on the response of both morphotypes to sub-lethal Cu stress, we performed tagRNA-seq on Cu-exposed and unexposed SW and ST cells grown in M2G and PYE medium. On average, 11,289,879 reads were generated for every sample. Of these reads, ca. 14.7 % were derived from the 5′ end of primary transcripts (transcription start sites, TSS), 17.7 % from the 5′ end of processed transcripts (processing sites, PSS), and the remainder of reads from transcripts not derived from 5′ ends (unassigned) (Supplementary Table S1). For most of the analyses, the different read libraries (TSS, PSS, and unassigned) were combined. This generated triplicates of eight data sets: two growth media (complex PYE medium and mineral M2G medium), two cell types (ST: stalked cells, SW: swarmer cells) and two conditions (Cu exposed and unexposed).

Principal component analysis (PCA) on all combined read libraries indicated, as expected, that the three main principal components correlated with culture medium, cell type and Cu stress, in decreasing order of explained variation (Figure 3). In addition, it is clear that the impact of Cu stress is more pronounced in M2G than in PYE, and for ST cells than SW cells. Nevertheless, the PCA indicates that a moderate Cu stress has a smaller impact on the transcriptome than cell differentiation or growth conditions.

The top-2000 genes with the highest variability in read coverage between all conditions were selected (out of a total of 4085 genes) for K-means clustering (Figure 4). This analysis showed that clusters 2, 4, 5, 6, 7, and 8 correlated with cell type (987 genes total), while clusters 1, 3, 9, 10, and 11 correlated with medium (596 genes total). Clusters 12, 13, 14, and 15 correlated with Cu stress (417 genes total). While it would be possible to discern gene-enriched pathways from these Cu stress-correlated clusters, we have opted to analyze and compare functional enrichment from all Cu-CT contrasts separately in order to attain a comprehensive analysis.

### 3.3. Transcriptomic Response of C. crescentus to Cu Stress

Differentially expressed genes were calculated for all Cu-CT contrasts, several of which were validated with qRT-PCR (Supplementary Figure S1). It is immediately clear that the transcriptomic response to Cu stress was more pronounced in mineral M2G than in the complex PYE medium, and in ST cells than in SW cells (Figure 5). ST cells in M2G medium showed the strongest transcriptional response to acute Cu stress. Out of 4085 genes, 1006 were differentially expressed and four (out of 20) eggNOG classes were enriched, i.e., class C (energy production and conversion), J (translation, ribosomal structure and biogenesis), O (posttranslational modification, protein turnover, chaperones) and V (defense mechanisms), with class O displaying the largest enrichment (Supplementary Table S2). The transcriptome of the SW cells in M2G medium was less strongly affected by Cu stress than their ST counterparts, and 270 out of 382 (71%) of up- or downregulated genes were also up- or downregulated in the ST cells (Figure 5). In complex PYE medium, the number of upregulated genes in ST cells was much higher than the number of downregulated genes, in response to Cu stress (Figure 5). In this contrast, only eggNOG class P, related to inorganic ion transport and metabolism, was overrepresented. SW cells in PYE medium were affected the least by Cu stress, and only class U, related to intracellular trafficking, secretion, and vesicular transport, was overrepresented. An overview of the functional roles of differentially expressed genes is shown in Table 1.

#### 3.3.1. A Core Gene-Set in the Response to Cu

Only 11 genes (9 of which are included in cluster L; see Figure 4) were upregulated by Cu exposure irrespective of cell type or growth medium (Table 2). The CCNA_03273,03362-03366 cluster encodes uncharacterized cytoplasmic, membrane and periplasmic proteins regulated by the extracytoplasmic function (ECF) sigma factor SigF (CCNA_03362) and the anti-sigma factor NrsF (CCNA_03273). In fact, the SigF regulon includes, in addition to this cluster, the two other clusters that were commonly upregulated by Cu exposure [41] (Table 2). Transcription of this regulon has already been shown to be upregulated in response to chromate (CrO_4_^2−^) and cadmium (Cd), in an oxidative stress-independent manner [25]. However, a *sigF* mutant displayed a similar sensitivity profile to these metals [41], but showed a severely impaired resistance to oxidative stress during the stationary phase [42].

In contrast to CrO_4_^2−^ and Cd^2+^, *sigF* inactivation severely impacted the growth of *C. crescentus* in the presence of Cu, demonstrating a role of this regulon in Cu tolerance (Figure 6). Interestingly, this cluster has also been studied in *Cupriavidus metallidurans* CH34, a model organism for metal resistance, in which it was first named *dax* in Monsieurs et al. (2011) [43] and renamed *gig* for “gold-induced genes” in Wiesemann et al. (2013) [44]. The cluster is upregulated in the presence of Au^3+^, Ag^+^ and Cu^2+^ [28,44]. In *C. metallidurans* CH34, it does not impact Cu resistance [44], but it should be noted that *C. metallidurans* CH34 carries multiple Cu resistance determinants that could mask its role.

The SigF-regulated CCNA_02999-03001 cluster codes for uncharacterized proteins, but CCNA_03000 and CCNA_03001 share 44.5% and 38.5% protein similarity with CCNA_03364 and CCNA_003363, respectively. Finally, the SigF-regulated CCNA_02834-02833 cluster is homologous to the MrsPQ system, which is involved in the protection of proteins from oxidative stress by repairing oxidized periplasmic proteins containing methionine sulfoxide residues [45]. Although, the SigF regulon plays a central role in the Cu response, expression levels were not similar across the different conditions. When exposed to Cu, expression in ST cells was higher than in SW cells when grown in PYE but not in M2G, and expression in M2G was higher than in PYE for both ST and SW cells. In unexposed conditions, the expression level of the SigF regulon did not significantly differ between cell types and growth medium.

The CCNA_00028 gene, encoding a TonB-dependent receptor, and CCNA_03372, encoding a bacterioferritin-associated ferredoxin, are both also upregulated under iron (Fe)-limiting conditions and repressed by Fur under Fe sufficiency [46], but their actual role is currently under investigation. However, as Fur has an important role in oxidative stress tolerance [46,47,48,49], their increased expression could be elicited in response to and to cope with oxidative stress generated by Cu.

#### 3.3.2. Oxidative Stress

Next to this core gene set, many genes upregulated by Cu stress in mineral M2G medium are involved in oxidative stress relief, a response that was not observed in complex PYE medium (Figure 7). This impact of ROS toxicity is reflected in the upregulation of catalase, two superoxide dismutases, and several hydroperoxide reductases, thioredoxins and glutaredoxins. In addition, the biosynthesis pathway of glutathione, an important antioxidant with a central role in cellular ROS protection, was strongly upregulated. OxyR, a major regulator of the oxidative stress response, was upregulated in both SW and ST cells. In *C. crescentus*, OxyR has been shown to control the expression of the catalase KatG and the alkyl hydroperoxidase system AhpCF [46,49]. Oxidative stress induction by Cu exposure has been described in several bacteria [28,50,51,52,53,54,55,56,57]. Interestingly, Cu shock, but not adaptation, was linked to the oxidative stress response in *Pseudomonas aeruginosa* [58].

The origin of Cu-induced oxidative stress is still a matter of debate [4]. While the cycling of Cu^+^ and Cu^2+^ has been linked to ROS formation, it has also been posited that the Cu-mediated destruction of enzymatic Fe-S clusters could result in the release of free Fe ions to the cell [11,12,59], which can cycle between divalent and trivalent forms to generate ROS via the Fenton reaction [9]. This mechanism of toxicity can also be observed in the oxidative stress response to non-redox-active metals such as Cd^2+^ [25]. Conversely, Fe starvation has also been linked to oxidative stress [60]. In this study, Cu stress had a notable impact on the expression of many Fe-S cluster-containing, redox-active enzymes such as cytochromes, cytochrome oxidases, and NADH quinone oxidoreductase components. However, with the current data, it is difficult to determine whether these enzymes are differentially regulated due to direct toxic effects of Cu, or due to their involvement in ROS detoxification. We note, however, that expression of the bacterioferritin Dps, which plays a role in oxidative stress response by storing free Fe ions [61], was not induced by Cu stress in any of the tested media or cell types. In addition, the bacterioferritin Bfr was downregulated during Cu stress, but only in ST cells in M2G medium. Consequently, the measurable effects of free Fe ions on oxidative stress seem relatively small. A final aspect of Cu-induced oxidative stress is evidenced by the upregulation of glutathione biosynthesis. Glutathione can reduce oxidized glutaredoxins, which serve as antioxidants. Both glutathione and thioredoxin have been shown to play a role in cell cycle regulation, and their concentrations vary between different growth phases [62,63]. However, Cu can also form a catalytic complex with glutathione, generating additional ROS instead of depleting them [64].

In PYE medium, no evidence of oxidative stress response was detected in either cell type, except for the upregulation of three sulfoxide reductase subunits in the ST cells, and two in the SW cells. We conclude that Cu exposure generates more oxidative stress in M2G than in PYE, despite comparable effects on growth. In addition, the oxidative stress response is more extensive in ST cells than in SW cells.

#### 3.3.3. Protein Misfolding and Proteome Rearrangement

A wide array of chaperones, such as DnaK, DnaJ and several of their homologs, were upregulated by Cu stress in M2G medium. Interestingly, while depletion of DnaK has been linked to an increase in RpoH abundance, both were strongly upregulated by Cu stress, indicating additional factors in their regulation. RpoH in turn has been linked to the control of heat shock chaperones, the upregulation of repair and maintenance functions, and the downregulation of growth and DNA replication [65,66]. The chaperones GroEL and GroES were upregulated by Cu exposure in M2G and have been associated with oxidative, saline, and osmotic stresses [67] (Figure 7). The dual-function holdase and thioredoxin oxidoreductase CnoX, which transfers unfolded proteins to DnaK/J and GroEL/ES, was also upregulated by Cu exposure in M2G [68]. In addition, general proteases such as the Clp, Hsl, and Lon complexes were upregulated. Cu and oxidative stress can lead to the production of toxic protein precursors and stress-denatured proteins [69,70]. The response to protein misfolding was also observed after general metal stress in *C. crescentus* [71]. Since proteotoxic stress can induce cell-cycle arrest, it is vital for the cell to overcome [72]. In this sense, it is interesting to note that the proteases FtsH and ClpXP are involved in regulatory processes and cell cycle progression [42,73,74,75,76,77]. In addition, the Lon protease allows *Caulobacter* cells undergoing proteolytic stress to maintain replicative ability [78]. It is also indirectly involved in cell cycle regulation since it can degrade the regulatory protein SciP [79]. Finally, we observed a general downregulation of translation, ribosomal structure and biosynthesis. This was evident from the observation that 79 genes out of 186 in eggNOG class J were downregulated, while only 7 were upregulated. Curiously, this observation was only made in ST cells in M2G medium, indicating again that this population is more responsive to Cu stress. In conclusion, these data illustrate a shift in the proteome and accumulation of misfolded proteins due to Cu stress.

In ST cells in PYE, several proteases were slightly overexpressed, indicating some level of proteome rearrangement. Two genes encoding homologs of the dual-function chaperone/protease DegP-family serine protease were upregulated, as well as a trypsin-like serine protease. No evidence for Cu stress-induced proteome rearrangement was detected in SW cells in PYE medium. As a whole, the proteome of *C. crescentus* cells was far less affected by Cu stress in PYE medium than in M2G medium.

#### 3.3.4. Metal Resistance and Transport Systems

Lawarée et al. (2016) [26] showed that the PcoAB system represents the main mechanism for Cu detoxification in *C. crescentus*. This system consists of the periplasmic Cu^+^ oxidase PcoA and the outer membrane Cu efflux pump PcoB. *pcoAB* transcription was observed during the SW-to-ST cell transition and was hardly induced by Cu in HIGG medium [26]. Seemingly, the induction of this system depends on both medium and cell type, as it was only induced by Cu stress in ST cells in M2G, but not at all in PYE.

*C. crescentus* also encodes additional metal tolerance clusters, some of which were induced by Cu stress. We measured a strong overexpression of the arsenic (As) resistance determinant ArsH and the arsenate reductase CCNA_01571. A similar upregulation of *ars* genes was observed after Cu exposure in *Cupriavidus metallidurans* CH34, but this phenomenon was not further analyzed [28]. However, some aspects of toxicity, such as ROS generation, are shared between Cu^2+^ and As^3+^ [80]. Such commonalities might also link the transcriptomic response to these metals. Likewise, upregulation of the tellurium (Te) resistance genes *terB*, *terC*, and CCNA_00755 was observed. Much like Cu^2+^ and As^3+^, Te^4+^ toxicity is characterized by oxidative stress [81]. While As and Te resistance determinants were induced in both cell types in M2G, the entire *czc*-like *nczCBA* cluster (CCNA_02471-02473) was induced by Cu stress only in ST cells in M2G. This gene cluster confers resistance to nickel and cobalt, and to a lesser degree to zinc (Zn) and Cd [82]. Likewise, the *czr* cluster (CCNA_02806-02811), consisting of an HME-RND export system and a P-type ATPase, was fully upregulated in ST cells in M2G. This cluster mainly confers resistance to Cd and Zn [82].

In agreement with Park et al. [83], the genes encoding the U/Zn/Cu responsive two-component regulatory UzcRS were induced by Cu stress in M2G. Although this regulatory system is responsive to U, Zn and Cu, it is not required for tolerance to these metals. Its regulon contains many genes involved in envelope stress response and several (antibiotic) transport systems, as well as a small regulatory RNA [83]. Indeed, 63 out of the 73 genes in the UzcRS regulon were differentially expressed in ST cells vs. 43 genes in SW cells. In addition, in M2G, transcription of the two-component system ChvGI, which is stimulated by starvation, prolonged DNA damage, acidic pH and cell wall stress, was also upregulated in response to Cu. ChvGI activates the ChvR sRNA (its expression was also upregulated with Cu), which in turn downregulates translation of the TonB-dependent receptor ChvT (its transcription was downregulated with Cu) [84]. Deletion of *chvT* has been shown to increase resistance to certain antibiotics (vancomycin and cefixime). Similarly, cross-regulation with many known and putative antimicrobial resistance mechanisms was observed in the Cu stress response. Toxicity mechanisms of organic antibiotics are often characterized by the induction of the oxidative stress response, similar to As and Te toxicity [85].

Finally, transport systems of several classes were differentially regulated due to Cu stress. In M2G medium, a relatively high percentage of ABC-transporters were upregulated, but in contrast a small percentage of TonB-dependent receptor proteins was affected by Cu stress. However, the substrates of many of these systems are not known, and consequently it is difficult to discover functional relations between their differential expression and their possible role in the Cu stress response. However, a special mention must be made of the downregulation of two ATP synthase systems. While co-import of Cu ions with the proton gradient could be a possible mechanism of toxicity, the downregulation of ATP synthases could result in a breakdown of the proton motive force as reported for silver toxicity, which is similar to Cu toxicity [86].

Curiously, the Cu-specific PcoAB system was not induced by Cu stress in PYE medium. In ST cells, the *ncz* and *czr* clusters (described above) were induced, as well as 6 TonB-dependent receptor proteins. All of these receptor proteins transport unknown substrates, except the hemin utilization system HutA. Receptor proteins such as these have been shown to play a role in metal acquisition, and as such may also be important transporters involved in the Cu stress response [87,88]. In SW cells in PYE, no metal resistance system was induced by Cu stress at all. This cell population displayed overexpression of the Type I secretion protein CdzB, which is part of a contact-dependent bacteriocin system [89], and a ferrous iron transport protein. The latter observation again highlights the role of Fe ion transport in the Cu stress response, apparently influential in both tested media.

#### 3.3.5. Amino Acid Metabolism

Several amino acid biosynthesis pathways were differentially regulated by Cu exposure. In ST cells, L-cysteine biosynthesis was strongly upregulated in M2G, similar to observations made in *Cupriavidus* [28,55]. In addition, both the pathway from 3-phosphoglycerate to serine and the assimilation of sulfate to hydrogen sulfide were upregulated, providing essential precursors for L-cysteine biosynthesis. At the same time, the cysteinyl-tRNA was downregulated and no significant change in the expression of cysteinyl-tRNA synthetase was observed, indicating a decreased consumption of cellular L-cysteine pools for protein synthesis. All the while, the synthesis pathways leading to methionine were downregulated. The essential role of thioether residues in Cu ion binding has been reviewed by Davis and O’Halloran (2008) [90]. We conclude that cellular sulfur pools seem to be geared towards L-cysteine synthesis, but that this cysteine is not used in translation so much as in the synthesis of other cysteine-containing compounds such as glutathione and thioredoxins. Curiously, the disparate use of S and cysteine pools was not as strongly observed in SW cells or in both morphotypes in PYE medium.

The biosynthesis of arginine was downregulated in both morphotypes in M2G medium. Conversely, in *Mycobacterium tuberculosis*, arginine biosynthesis was upregulated by oxidative stress, and arginine deprivation was linked to antioxidant depletion [91]. In *Pseudomonas putida* KT2440, arginine-derived polyamines were shown to play a role in oxidative stress relief [92]. Finally, arginine exacerbated oxidative stress from hydrogen peroxide addition in *Streptococcus mutans* [93]. In this *S. mutans* study, a metal transporter was implicated in the observed behavior, among other genes. All in all, the role of arginine in Cu homeostasis seems to be complex, and we cannot provide a final explanation for the observed behavior without additional experiments.

In ST cells, grown in PYE medium, we observed an overall downregulation of the complete histidine degradation pathway, suggesting an increased need for L-histidine. Similar to cysteine, histidine residues have been implicated as a cellular Cu ligand [90,94]. This property has been utilized to engineer *C. crescentus* strains for biosorption of Cd^2+^ ions, which have similar binding characteristics to Cu^2+^ [95]. While the specific role of histidine in the Cu stress response has not been studied in bacteria, in the fungal pathogen *Aspergillus fumigatus*, a strong relation between histidine biosynthesis and metal resistance has been shown [96]. Some overexpression of sulfate assimilation genes, possibly integrating into L-cysteine metabolism, was also noted.

#### 3.3.6. Regulation by Small Regulatory RNAs

**Figure 8:**
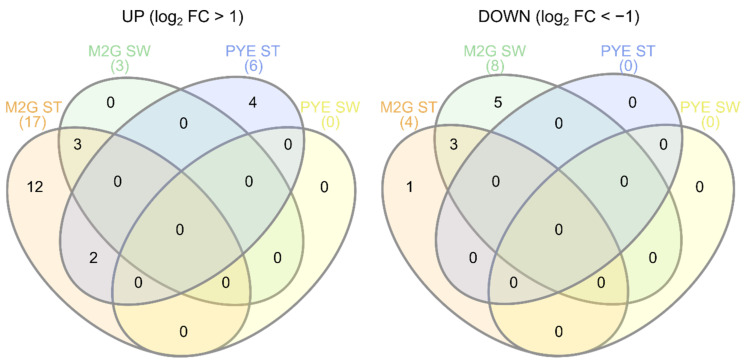
Venn diagrams showing overlap between sRNAs upregulated (**left**) and downregulated (**right**) by Cu stress in all experimental conditions.

*C. crescentus* harbors many small regulatory RNAs (sRNAs) that can influence gene expression at the post-transcriptional level in a variety of ways, such as direct interaction with mRNAs [97,98]. Differential expression of sRNAs in response to Cu was observed in all conditions, except for SW cells in PYE. The strongest evidence for sRNA regulation in response to Cu stress was detected in M2G medium (17 upregulated in ST cells, 3 up- and 1 downregulated in SW cells). In PYE medium, six sRNAs were upregulated in ST cells (Figure 8). The ChvR sRNA and its function have been described in Section 3.3.3, but unfortunately the target genes have yet to be identified for most other sRNAs. Therefore, we predicted putative target genes for all differentially expressed sRNAs for which no experimentally validated target has been reported (Supplementary Table S3). Several of these sRNAs could be involved in the regulation of proteins relevant to the Cu stress response, such as transporters, regulators, and superoxide dismutases. In conclusion, while regulation via sRNAs is potentially extensive and far-reaching, there is a need for additional experiments to determine sRNA-target interactions. An overview of computational and experimental validation strategies can be found in Georg et al. (2020) [99].

### 3.4. Role of Environmental Conditions in the Cu Stress Response

It is clear that the Cu stress response of SW cells in PYE is more closely related to that of ST cells in PYE than to that of SW cells in M2G medium (Figure 4). Similarly, the response of SW cells in M2G medium aligns more closely to that of ST cells in M2G than to that of SW cells in PYE medium. The differences in the response to Cu stress in the mineral medium M2G and the complex medium PYE can be at least partially ascribed to the speciation of Cu ions in these media. While a modelling approach might provide insights into approximate speciation at chemical equilibrium, the addition of highly complex, living bacterial cells would generate a large degree of uncertainty. Indeed, the important role of medium composition on the free metal ion concentration, which lies at the basis of metal toxicity [100], has been shown in several studies [101,102]. To illustrate, there can be a difference of at least 3 orders of magnitude in measured free Cu ion concentrations between complex and defined media, at relevant levels of Cu supplementation [102]. Here, we will provide a brief overview of the main chemical species suspected to sequester Cu ions. Nies (2016) compared the main chelating agents of transition metals in mineral and complex media [103]. M2G medium contains ca. 20 mM of phosphate anions, as well as 8 µM of EDTA. These compounds are the most likely to sequester supplemented Cu. However, Fe^3+^ will outcompete Cu^2+^ for EDTA binding, since the stability constant of Fe^3+^-EDTA (logK = 25 [104]) is higher than that of Cu^2+^-EDTA (logK = 18.46 [105]). Even though 15 µM of CuSO_4_ was added to the medium, which contains only 10 µM Fe^3+^, it is unlikely that EDTA is able to sequester much of the Cu ions present. Consequently, the extant phosphate moieties are the most likely Cu ligands. It should be noted that a far-reaching impact of Cu addition on the cells’ phosphate metabolism is not expected due to the high PO_4_^3−^ concentration relative to Cu in the medium. The sulfate and chloride anions are fairly ‘hard’ ions, and are thus unlikely to sequester the relatively ‘soft’ Cu^2+^ ions. PYE medium contains 2 g of peptone and 1 g of yeast extract per liter of medium. Adapting the calculations of Nies (2016) [103] and Sezonov et al. (2007) [106], this leads to final concentrations of 44 µM cysteine and 396 µM histidine from peptone, as well as 23 µM glutathione (containing one cysteine residue per molecule) from yeast extract. Cysteine and histidine represent the main transition metal-sequestering amino acids in this composition [103]. Since we added 175 µM Cu, enough binding capacity exists in the form of cysteine and histidine residues alone for the complexation of essentially all added Cu. However, we must be cautious in the interpretation of these results, since we do observe moderate toxicity after the addition of CuSO_4_ to PYE medium. First, other amino acid residues may also sequester Cu, albeit with lower stability constants. As the sum of these residues is present in higher concentrations than cysteine and histidine, they may still play important parts in the final equation [106]. In addition, casein hydrolysates from different sources can be quite different in composition, which has been shown to affect the oxidative stress response [107]. Finally, we have not taken the intrinsic Cu-binding capacity of the bacterial cell population into account. At an inoculum density of 4 × 10^8^ cells per ml, it is likely that a relevant fraction of available Cu is associated with the bacterial cell surface.

As mentioned above, it is difficult to assess the total Cu-binding capacity in the presence of living cells. However, it is clear that more CuSO_4_ must be added to PYE medium than M2G medium to achieve a similar deleterious effect on *C. crescentus* growth (Figure 2). Even with a higher Cu concentration in PYE, the transcriptomic response of SW and ST cells to Cu stress in PYE was markedly weaker than that of their counterparts in M2G medium. In addition, these disparate responses showed clear differences. No oxidative stress response was observed in PYE medium, while it was highly evident in M2G medium. This could be due to the presence of readily available glutathione and other antioxidants derived from the yeast extract in PYE [108]. The proteomic reconfiguration, which was also only observed in M2G medium, would then be a consequence of the oxidative stress in this medium. Interestingly, we detected changes in amino acid metabolism regulation in both media. In M2G medium, cysteine and arginine anabolism was overexpressed. As M2G does not contain amino acids and they must all be biosynthesized by the cell, we conclude that cysteine and arginine actually play a role in the Cu stress response. In contrast, in PYE medium, a need for histidine was observed after Cu stress. While histidine has been implicated in metal resistance, it could be the case that Cu-ligated histidine in the medium cannot (easily) interact with its cognate cellular importer, thus creating an apparent need for this amino acid. In other words, histidine could be ‘titrated away’ from cellular uptake by interaction with Cu. We did not find any indication of a similar phenomenon regarding cysteine, but then the cellular requirement for cysteine relative to its environmental concentration might be lower than is the case for histidine. However, to our knowledge, no such indirect toxicity mechanism has been described in literature.

We conclude that the mechanisms of Cu toxicity show extensive differences between the mineral medium M2G and the complex medium PYE. This is likely in large part due to the chemical speciation of Cu ions in these media. In addition, both cell types display intrinsic differences between their transcriptomes in M2G and PYE, mostly evident in their amino acid and carbohydrate metabolisms (as further shown in Section 3.5). Since cells must import either mineral compounds and glucose (in M2G) or complex organic compounds such as amino acids (in PYE), the constitutive transportomes of cells in these media could play a crucial part in their intrinsic ability to handle the sudden addition of CuSO_4_. In addition, both phosphates and peptone components can alter the cell surface affinity for metal ions [109]. However, other than on the transcriptomic level, little study has gone into the phenotypes of *C. crescentus* in disparate culture media [110]. Finally, the diverse real-world environments where *Caulobacter* species are isolated are not perfectly mirrored by either M2G or PYE medium, so aspects from the Cu stress responses from either medium could be relevant in these diverse situations. For example, in the oligotrophic freshwater streams and lakes where *Caulobacter* species are commonly isolated [111], an oxidative stress response would be a likely consequence of Cu stress (as seen in the mineral M2G medium). Conversely, for *Caulobacter* species found in soil habitats [112], where the concentration of complexing compounds is higher, the Cu stress response could align more closely with the reorganization of the transportome observed in the complex PYE medium.

### 3.5. Cu Stress Perception by Stalked and Swarmer Cells

Overall, the differentially expressed genes in the swarmer cell population belonged to the same functional groups as in the ST cell population. However, fewer genes in each SW cell population were differentially expressed (Figure 5). Generally, the genes more strongly up- or downregulated between the Cu-stressed and control ST cell populations are those detected to be up- or downregulated in the SW cell population. The few systems exclusively altered by Cu stress in SW cells point to a slightly altered use of sulfate, phosphate and nitrogen sources, but a link to Cu detoxification is not clear. The question then remains: why are SW cells impacted to a lesser extent than ST cells? While it is evident that the number of genes upregulated due to Cu stress is larger in ST cells than in SW cells, many of the detoxification systems induced in ST cells could have already been active in SW cells. The constitutive expression of such systems becomes apparent when comparing the control conditions of ST and SW cells. In M2G medium, it is striking that of the 734 genes differentially expressed by Cu stress in ST cells but not differentially expressed in SW cells, 268 were differentially expressed between the control conditions of ST and SW cells. Of these 268 genes, 196 were overexpressed in control-condition SW cells. Among these genes were many relevant cell defense systems, including chaperones such as GroES/EL and DnaK/J, parts of cysteine and glutathione metabolism, the catalase KatG, and metal resistance mechanisms such as PcoAB. In previous studies, it has been shown that the intracellular redox state is dependent on the growth phase in *C. crescentus*, with a more reducing state in SW cells and a more oxidizing state in ST cells [63,113]. This relatively reducing state in SW cells could be linked to the upregulation of cell protection systems, but the causality of this relationship is unclear. We note that oxidative stress represents but one aspect of Cu stress, and little information has been gathered on the cell cycle dependency of other aspects such as the accumulation of toxic protein precursors or shifts in amino acid requirements. Finally, we have previously shown that SW cells accumulate more Cu than ST cells in order to sustain a negative chemotaxis to flee from the Cu stress [26]. This is consistent with the less extensive induction of cell defense mechanisms observed in the current study. In SW cells in PYE medium, a similar high basal expression level was noted for several systems induced by Cu stress in ST cells, including several transport systems. We conclude that in both media, the SW morphotype is less responsive to Cu stress, which might be due to the inherently higher level of mRNAs encoding cell protection systems.

We must mention that 112 genes were differentially regulated by Cu stress solely in the SW cells in M2G medium. Downregulation of the regulatory proteins of the nitrogen metabolism, such GlnB, GlnK, and NtrC, might indicate a different use, need, or level of assimilation of N sources under Cu stress. Genes encoding the phosphate transporters PstA and PstC as well as the P-starvation inducible protein PhoH were downregulated. In addition, several genes involved in S transport were downregulated. We also observed a slightly altered expression of genes involved in flagellar biosynthesis and motility, such as *fliQ*, *flbT*, and *cheW*. In addition, genes involved in stalk biogenesis *cpaA* and *hfaE* were differentially regulated. However, these observations were more diffuse than the overall tendency towards an oxidative stress response and other systems similarly induced in the ST cells.

### 3.6. Transcription Start Site Analysis

**Figure 9:**
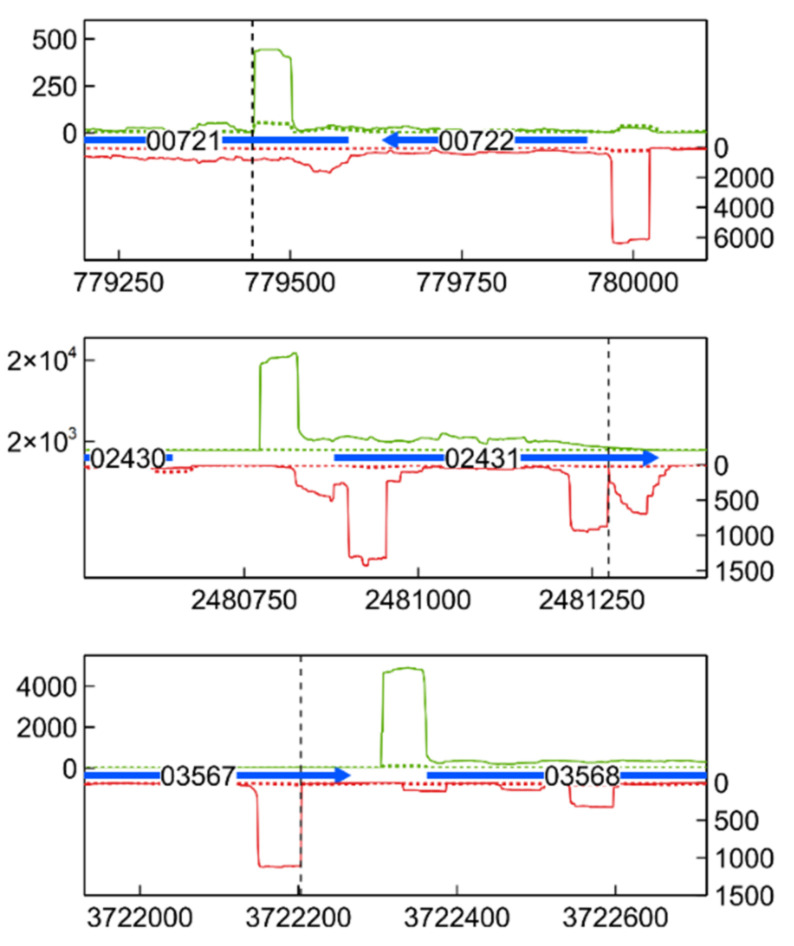
Transcription profile of identified aTSSs for selected genes. Combined TSS read counts of the three biological replicates of Cu-exposed (solid line) and unexposed (dotted line) stalked cells in M2G medium are shown for the positive (green) and negative (red) strand. Coding sequences are represented by a horizontal blue arrow (number is CCNA_ locus tag, x axis positions being the genomic location). Detected aTSSs are indicated with a vertical dashed line.

**Figure 10:**
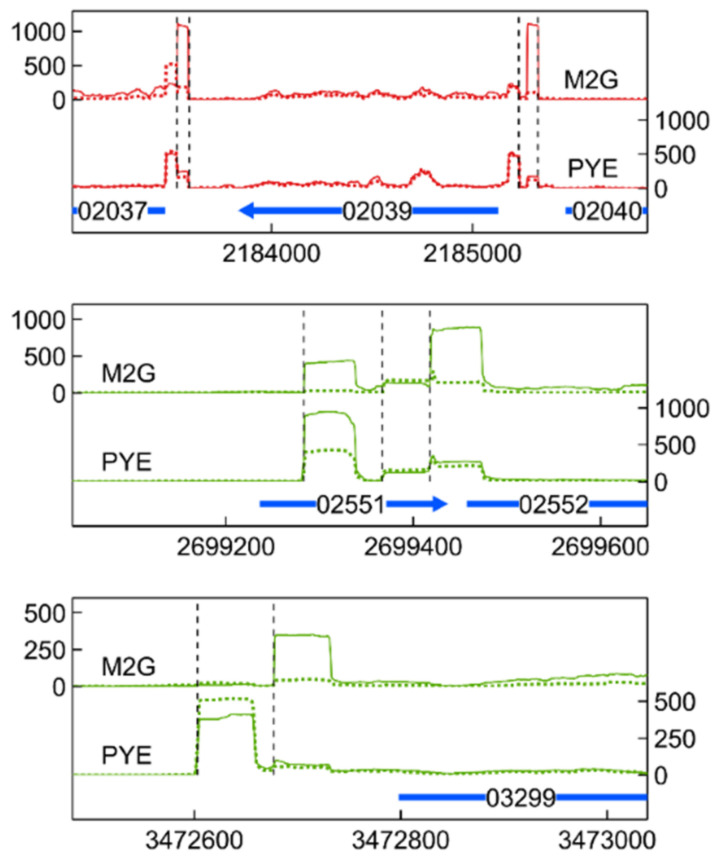
Transcription profile of identified alternative TSSs for selected genes. Combined TSS read counts of the three biological replicates of Cu-exposed (solid line) and unexposed (dotted line) stalked cells in M2G (upper) and PYE (lower) medium are shown for the positive (green) and negative (red) strand. Coding sequences are represented by a horizontal blue arrow (number is CCNA_ locus tag, x axis positions being the genomic location). Detected TSSs are indicated with a vertical dashed line.

The tagRNA-seq protocol used in this study allowed us to accurately detect 5′ ends of primary RNAs [28]. Several previous studies have performed genome-wide transcription site (TSS) analyses, leading to a high degree of accurately defined TSSs in standard growth conditions [114,115,116,117]. Consequently, we have opted to focus on the detection of new TSSs in the Cu stress conditions (Figure 9, Supplementary Table S5). In addition, several genes induced by Cu stress seem to be transcribed from multiple TSSs, indicating complex stress-dependent regulation (Figure 10, Supplementary Table S6).

Newly detected TSSs were often associated with known CDSs, with transcription proceeding either in the same sense (intragenic, iTSS) or in the antisense direction (aTSS). iTSSs, located inside coding regions, can mark the start of alternative mRNAs encoding shortened proteins. Alternatively, they can indicate trans-acting, non-coding RNA. Such iTSSs were found in the genes encoding the S-layer structural protein RsaA (not to be confused with the sRNA RsaA in *Staphylococcus aureus* [118]), and the sRNA CCNA_R0092. While expression of the *rsaA* gene itself was not induced by Cu stress, increased expression of its iTSS was detected after Cu exposure in M2G medium. Sorption of metals onto S-layer proteins has been shown in other bacteria [119,120], and it is possible that an alternate, shorter RsaA protein has disparate Cu-binding characteristics, putatively playing a part in the Cu stress response. Since the role of the sRNA CCNA_R0092 is not known, we cannot comment on a possible role of its iTSS.

Antisense TSSs are found on the antisense strand of a coding region, and usually denote the start of cis-acting sRNAs, which can form an important part of post-transcriptional regulation [121]. Some of these aTSSs were found in CDSs induced by Cu stress, such as GroEL (CCNA_00721), the Hsp20-family gene CCNA_02341 (3 distinct aTSSs), the TonB-dependent receptor protein CCNA_001738 (unknown substrate), and the acyl-CoA dehydrogenase CCNA_03567 (Figure 9 and Supplementary Table S5). All aTSSs linked to these CDSs were induced by Cu stress. Finally, several aTSSs were induced by Cu stress, while their corresponding CDSs were not. The PilA mRNA, encoding a Type IV pilin, was present at higher levels in SW cells than in ST cells, but was not induced by Cu stress. However, a Cu stress-inducible aTSS was detected on its antisense strand. The PilA protein was recently shown to couple mechanical cell-surface contact with initiation of the cell cycle [122]. Expression of *pilA* is regulated by the cell-cycle regulator CtrA [123]; nonetheless, non-canonical factors governing its expression seem to be present.

Finally, several genes seemed to be transcribed from multiple TSSs, indicating complex regulation with multiple regulators. Such alternative TSSs were found for genes encoding transport systems, such as the TolC-family outer membrane protein CCNA_03299 and the ABC transport ATP-binding cassette CCNA_03483. In the case of CCNA_03299, a clearly medium-dependent alternative TSS was detected. Unfortunately, the substrates of these systems are not known, but both systems are induced by Cu stress in M2G. In addition, alternative TSSs were linked to genes encoding proteases and chaperones such as *lon* (CCNA_02037), *clpX* (CCNA_02039), *clpS* (CCNA_02552), and the gene encoding the Hsp20-family protein CCNA_03706 (Figure 10 and Supplementary Table S6). For all of these genes, at least one TSS was previously detected (i.e., Zhou et al., 2015 [117]). It is likely that additional TSSs, often preferred during Cu stress, can give rise to mRNA isoforms. The biological relevance of these isoforms remains to be studied. In any case, the clear dependency of transcription start site selection on environmental conditions shows that the response to Cu stress is governed by different regulators in these conditions.

### 3.7. Correspondence with Previous Transcriptomic Studies

Although cross-study transcriptome results are difficult to compare due to variability in, e.g., experimental setup, measurement sensitivity, and statistical methods, we examined the correlation of our results with existing datasets.

Hottes et al. [110] used a microarray analysis to investigate the transcriptome of unsynchronized *C. crescentus* populations in PYE and M2G medium. Both datasets can only be partly compared, because of limited data and data analysis availability. Nevertheless, based on eggNOG class overrepresentation (amino acid metabolism, signal transduction, carbohydrate metabolism, motility, inorganic ion transport and metabolism, and nucleotide metabolism), both studies correspond well (Supplementary Table S2).

The transcriptomes of synchronized *C. crescentus* populations at several points along the cell cycle were studied by Fang et al. (2013) [124]. We compared their and our (without CuSO_4_) dataset, and for SW cells, 96 of their top 100 upregulated genes in SW cells were also upregulated in our data, in both M2G and PYE medium. Similarly, of the top 100 upregulated genes in ST cells, 99 were upregulated in ST cells in our data, again in both tested media. Thus, we conclude that the results of our cell synchronization corresponded very closely to those reported in literature.

To validate the detection of transcription start sites (TSSs), we compared the list of detected TSSs to those of a similar study. In Zhou et al. (2015) [117], TSSs were detected from RNA extracted at several points along the cell cycle of *C. crescentus*, in M2G medium, with an RNA-Seq-based method. We examined the overlap for both SW cells and ST cells in M2G medium. Around 35% of the 2728 TSSs reported in Zhou et al. were also detected in our study, for both cell types. The discrepancy can largely be attributed to differences in RNA treatment and processing, as well as the software used to handle sequencing reads and TSS identification. This is evident from the fact that we detect higher percentages of TSSs experimentally validated by, e.g., β-galactosidase reporter assays in both cell types (56.3 % in SW cells, 49.4 % in ST cells). Finally, Zhou et al. considered TSSs from RNAs expressed at eight time points in the cell cycle, while we only have data from two time points. In conclusion, our results correlate fairly closely to those reported previously.

## 4. Conclusions

We studied the transcriptomes of Cu-stressed *C. crescentus* SW and ST cell morphotypes in a defined mineral (M2G) and a complex (PYE) medium, in order to examine the influence of disparate cellular environments on the genome-wide Cu stress response. While the level of Cu supplementation achieved similar deleterious effects in both media, a far larger impact on the bacterial transcriptome was observed in M2G. In parallel, more extensive changes to the transcriptome of ST cells, relative to SW cells, were found in both media. The ECF sigma factor SigF and its regulon, containing several genes of unknown function, were induced by Cu stress in all tested conditions. In addition, disruption of *sigF* induced a Cu-sensitive phenotype, highlighting the importance of this regulon in the Cu stress response. In M2G, both morphotypes displayed a strong oxidative stress response, as well as a proteome rearrangement likely because, in part, of the accumulation of toxic pre-proteins. Several metal resistance mechanisms were induced in both media, but the PcoAB system, which confers resistance to Cu, was only upregulated in stalked cells in M2G. While a role for cysteine and arginine metabolisms could be discerned in the stress response in M2G, there was an apparent need for histidine in PYE. In general, the response to Cu exposure depended greatly on the environment. While neither of the tested media necessarily directly represent natural environments, it is clear that we must be careful in generalizing data from one environment to the other. Further validation of the role of specific resistance mechanisms is largely out of the scope of this manuscript, but these results emphasize the importance of tailoring the experimental conditions to the biological question at hand. Several questions remain open, such as the precise interactions between cell (surface), medium components and Cu ions, which could be further unraveled by a combinatory approach, including transcriptomics, proteomics, a direct assessment of the chemical speciation and dynamic modelling. In addition, it would be interesting to modulate the cell phenotype before applying Cu stress, e.g., by pre-inducing Cu resistance mechanisms, or applying stress to cells in stationary phase or encapsulated in biofilms, which are often more relevant than the exponential-phase populations used in this study. Finally, neither M2G nor PYE are especially oligotrophic, and consequently the response to Cu stress in freshwater or relevant soil extracts could be the object of further study. To a lesser extent, the Cu stress response was dependent on the morphotype, and we found that many relevant resistance systems were constitutively expressed in SW cells, indicating a higher level of inherent metal tolerance. Further study of the role of the morphotype could reveal the ecological significance of the more resistant SW cells. Nevertheless, it is clear that the response to Cu stress varies strongly between culture media and cell type, which shows the variety of ways in which a population of cells with the same genome can handle such a stress.

## Figures and Tables

**Table 1:** Overview of functional roles of differentially regulated genes in all Cu-CT contrasts.

Pathway or Functional Relation	Specific Pathway	M2G ST		M2G SW		PYE ST		PYE SW	
		Up	Down	Up	Down	Up	Down	Up	Down
Metal resistance mechanisms		19	0	5	0	6	1	0	0
Proteases and peptidases		32	9	13	6	4	0	0	0
Ribosome synthesis		2	78	0	24	0	0	0	0
ATP synthesis		0	10	0	6	0	0	0	0
sRNAs		16	0	3	1	5	1	0	0
Antibiotic resistance		12	3	5	1	0	0	0	0
Chaperons		13	2	5	2	0	0	0	0
Transporters	ABC transport	18	4	9	3	1	0	0	0
	TonB-dependent receptors	6	10	2	4	4	2	1	0
	Other	19	19	2	9	4	0	3	0
Regulators	Known function	19	19	6	13	6	2	1	0
	Other	33	16	11	3	4	0	1	0
Amino acid metabolism	Methionine biosynthesis	0	4	0	0	0	0	0	0
	Cysteine biosynthesis & S assimilation	8	2	0	0	1	3	0	0
	Arginine biosynthesis	1	7	1	5	0	0	0	0
	Serine biosynthesis	2	7	1	1	0	0	0	0
	Histidine	2	1	0	0	4	0	1	0
Redox and oxidative stress-related	Radical removal & antioxidants	16	2	11	0	4	0	2	0
	Glutathione cycle	7	2	2	1	0	0	0	0
	Other	37	39	14	5	7	0	2	0
Hypothetical proteins		73	33	48	23	22	2	2	0

**Table 2:** List of genes upregulated by Cu stress in all tested conditions and cell types.

Locus Tag ^1^	Gene Product	Log2 Fold Change			
		M2G		PYE	
		ST	SW	ST	SW
00028 ^$^	TonB-dependent receptor protein	1.85	1.87	4.64	2.26
02833	Sulfoxide reductase heme-binding subunit	5.32	4.18	3.13	1.84
02834	Sulfoxide reductase catalytic subunit	5.84	5.73	3.34	2.60
02999	DNA-binding domain-containing protein	5.03	4.32	3.24	2.04
03000	DUF692 domain-containing protein	5.94	5.77	3.63	2.80
03001	Hypothetical protein	5.67	6.21	3.17	2.00
03273	Anti-sigma factor NrsF	4.15	4.27	2.88	1.84 *
03362	ECF-family sigma factor SigF	4.44	4.70	2.78	2.17
03363	DUF2282 domain-containing protein	5.79	6.07	3.46	2.01
03364	DUF692 domain-containing protein	6.16	6.31	4.22	3.53
03365	Hypothetical protein	5.86	5.06	4.15	2.74
03366	DoxX-family protein	5.50	4.35	4.27	1.40 *
03372 ^$^	Bacterioferritin-associated ferredoxin	4.50	5.59	2.97	5.33

^1^ Locus tag is preceded by CCNA_. ^$^ All genes except those marked with $ are under control of SigF. * Only significant (*p* < 0.05) on the basis of statistical testing without *treat* lfc cutoff (see Section 2).

## Data Availability

RNA sequencing data are available within the Sequencing Read Archive (SRA) of NCBI using the accession number PRJNA721587.

## Supplementary Materials

The following are available online at https://www.mdpi.com/article/10.3390/microorganisms9061116/s1, Figure S1: Differential expression of 4 selected genes, in stalked cells (ST) and swarmer cells (SW) in 2 culture media (M2G and PYE). Bars represent log2 fold change values from qRT-PCR data, with diamonds representing log2 fold changes calculated with tagRNA-seq. CCNA accession numbers are shown at the bottom. Table S1: Raw read counts of tagRNA-seq libraries, Table S2: Overrepresented eggNOG classes calculated from the differential expression analysis of several tagRNA-seq contrasts. Table S3: Differentially expressed small regulatory RNAs and their top 3 putative target genes, based on maximal binding free energy. Table S4: Primers used for qPCR. Table S5: Positions of Cu stress-induced intragenic and antisense TSSs. Log2 fold change due to Cu stress in disparate experimental conditions, as well as genomic context, is shown for each TSS. Previously known TSSs are shown in bold. Table S6: Genes putatively transcribed from multiple TSSs and their corresponding alternative TSS positions. Previously known TSSs are shown in bold. Excel file: Sheet 1: Overview of file contents and nomenclature of headers. Sheet 2: Differential gene expression of all studied contrasts, including log2 fold change, false discovery rate, and gene eggNOG classes. Sheet 3: List of detected transcription start sites and their coverage (before normalization). Sheet 4: Overview of functional classification of differentially expressed gene annotations.
