# Supplementary material for: Environmental Conditions Modulate the Transcriptomic Response of Both Caulobacter crescentus Morphotypes to Cu Stress
Source: Microorganisms. 2021 May 21;9(6):1116. doi: 10.3390/microorganisms9061116 (PMC8224329; doi:10.3390/microorganisms9061116)
Supplement: Supplementary file 1 [file microorganisms-09-01116-s001.zip › microorganisms-1217387-supplementary/supplementary data/Supplementary_materials.pdf]

microorganisms

Supplementary Materials

Supplementary Table S1. Raw read counts of tagRNA-Seq libraries.

Sample				Unassigned reads	TSS reads	PSS reads	Total reads
M2G	St	Cu	1	7029956	941625	1996589	9968170
			2	6493672	772038	1343006	8608716
			3	5637679	804827	1432578	7875084
		Ct	1	6555009	742017	1496397	8793423
			2	7052540	800345	1684963	9537848
			3	6574748	801229	1740855	9116832
	Sw	Cu	1	8011110	1100154	2198595	11309859
			2	6211153	909899	1597453	8718505
			3	5336313	685777	1557530	7579620
		Ct	1	6221880	684872	1911990	8818742
			2	6651033	840671	1689593	9181297
			3	6777805	572727	1195243	8545775
PYE	St	Cu	1	7700765	2759732	1875608	12336105
			2	8225721	2959976	2319953	13505650
			3	8770754	2715491	2418996	13905241
		Ct	1	9130811	2447386	2087056	13665253
			2	8637565	2408231	2113804	13159600
			3	8409195	2472966	2020761	12902922
	Sw	Cu	1	8585751	3735415	2590338	14911504
			2	8272520	3076795	2754637	14103952
			3	8643418	2838859	2561540	14043817
		Ct	1	8530708	2490665	2371096	13392469
			2	7731070	2765604	2252039	12748712
			3	8850095	2783986	2593916	14227997

Supplementary Table S2: Overrepresented eggNOG classes calculated from the differential expression analysis of several tagRNA-seq contrasts.

Contrast	Overrepresented eggNOG class	Class content	p-value
MStCu-MStCt	O	Posttranslational modification, protein turnover, chaperones	$5.98*10^{-104}$
	J	Translation, ribosomal structure and biogenesis	$6.49*10^{-28}$
	C	Energy production and conversion	$7.26*10^{-5}$
	V	Defense mechanisms	$2.58*10^{-3}$
MSwCu-MSwCt	O	Posttranslational modification, protein turnover, chaperones	$3.29*10^{-3}$
	V	Defense mechanisms	$1.99*10^{-2}$
PStCu-PStCt	P	Inorganic ion transport and metabolism	$3.13*10^{-8}$
PSwCu-PSwCt	U	Intracellular trafficking, secretion, and vesicular transport	$6.11*10^{-3}$
MCt-PCt	E	Amino acid transport and metabolism	$9.84*10^{-36}$
	T	Signal transduction mechanisms	$7.33*10^{-14}$
	G	Carbohydrate transport and metabolism	$8.04*10^{-5}$
	N	Cell motility	$2.02*10^{-2}$
	P	Inorganic ion transport and metabolism	$3.65*10^{-2}$
	F	Nucleotide transport and metabolism	$4.77*10^{-2}$

Supplementary Table S3. Differentially expressed small regulatory RNAs and their top 3 putative target genes, based on maximal binding free energy.

sRNA	Log2(FC) of Cu-Ct				Target gene accession[1]	Gene product	Binding site[2]
	M2G St	M2G Sw	PYE St	PYE Sw			
R0002	4.65	0.30	0.59	0.33	01301	NAD dependent formate dehydrogenase alpha subunit	2038><2108
					02875	Hypothetical protein	55><81
					02651	Drug/metabolite exporter	-188><-160
R0032					02892	Hypothetical protein	-15><3
					01081	Hypothetical protein	38><58
					03699	M16 falily peptidase	2538><2554
R0078					03523	Hypothetical protein	-153><-124
					00723	Glyoxalase family protein	138><159
					02370	TonB-dependent maltose transporter MalA	1822><1850
R0088	3.79	1.35	1.74	-0.10	03779	Cytosolic protein	-179><-150
					02816	Hypothetical protein	130><147
					02454	N-methylhydantoinase (ATP hydrolyzing)	3640><3657

R0092					02816	Hypothetical protein	121><144
					00664	C4 dicarboxylate transport protein	45><67
					02642	UDP-N-acetylmuramoylalanyl-D-glutamate-2,6-diaminopimelate ligase	196><211
R0095					00006	Enoyl-CoA hydratase	271><789
R0097	0.82	0.66	4.19	0.51	02483	Acyl-CoA synthetase	-34><-24
					00739	Hypothetical protein	55><65
					03872	tRNA modification GTPase TrmE	1053><1061
R0100[3]	3.12	1.21	0.22	0.37	03108	TonB-dependent receptor	-70><-59 & -20><-10
R0110	1.18	0.67	1.80	-0.01	00546	Hypothetical protein	-195><-166
					00545	Acetoacetyl-CoA reductase	651><680
					00817	L-sorbosone dehydrogenase	1299><1324
R0111					00593	Hypothetical protein	1177><1584
R0119	3.87	2.68	0.52	1.17	01083	Hypothetical protein	-110><-86
					00275	Transposase	-167><-146
					01895	MFS superfamily transporter	1244><1258
R0128	1.37	0.78	2.74	-0.03	01034	TonB-dependent outer membrane receptor	-28><1
					00423	Phosphohydrolase	-110><-85
					03204	Alanine racemase	-96><-74
R0131					00697	Hypothetical protein	300><321
					02509	Glycosyltransferase HfsG	594><613
					02270	MarR-family transcriptional regulator	-20><-4
R0134	3.18	-0.88	1.56	0.59	01174	LysR family transcriptional regulator	-96><-68
					00181	Type II secretion pathway protein M	-158><-143
					00471	GDP L-fucose synthase	-188><-172
R0140	1.44	0.64	0.41	0.52	02329	Exodeoxyribonuclease VII large subunit	-110><-89
					00771	Hypothetical protein	89><118
					00798	CydC ATP-binding protein (ABC transport)	-68><-43
R0143					01584	Multimodular transglycosylase	-169><-142
					02235	Putative secreted protein	-75><-47
					00989	Acyl-esterase	11><26
R0158	1.39	-0.28	7.85	0.02	02221	Methionine synthase I MetH	496><507
					01382	Long chain fatty acid-CoA ligase	1432><1448
					00056	Ribosomal protein S18 acetyltransferase	410><426
R0161	3.26	0.17	3.01	0.38	03167	Hypothetical protein	469><492
					01215	Histidine triad protein	388><401
					03168	Hypothetical protein	517><537
R0162					02324	Hsp33-like chaperonin	-176><-148
					01651	DNA gyrase subunit A	-186><-161
					00105	MarC-family integral membrane protein	-151><-124
R0169	1.69	0.86	1.54	0.00	02606	Hybrid histidine kinase/receiver domain protein	842><871

					01515	C4 dicarboxylate transporter large subunit	633><659
					01604	Hypothetical protein	52><73
R0182	2.85	3.32	0.48	0.15	02949	Hypothetical protein	-167><-140
					03128	Drug/metabolite exporter	-53><-25
					00276	Hypothetical protein	274><298
R0199	4.93	1.27	1.81	0.04	02024	NADH dehydrogenase I subunit F	-83><-58
					02172	Transporter	1323><1350
					01650	Cu/Zn superoxide dismutase	8><24

Supplementary Table S4. Primers used for qPCR.

Name	Sequence (5′ → 3′)
mreB_F	CATCGAGGCCAAGCAGATGC
mreB_R	AGCCCTTGCGGTTGTGAACC
pcoB_F	CGCCCTATTGGTTCGAGCTG
pcoB_R	GTTGCAGGATCAGCCTCTGG
CCNA_00028_F	ATAGTTGGTGAACGCCCAGG
CCNA_00028_R	ACGATCGCTACGAGAGCAAG
CCNA_00140_F	GATGGATCCCATCCTGGGGA
CCNA_00140_R	AGAACCACAGCTTATGGCCC
CCNA_01010_F	CCAGGACCCCTACTGCCT
CCNA_01010_R	GAAGATCAGCGGATTGTCCGA

Supplementary Table S5: Positions of Cu^{2+} stress-induced intragenic and antisense TSSs. Log2-fold change due to Cu^{2+} stress in disparate experimental conditions, as well as genomic context are shown for each TSS. Previously known TSSs are shown in bold.

5′ end position	Putative 3′ end position	Strand	M2G St induction	M2G Sw induction	PYE St induction	PYE Sw induction	Genomic context
779445		+	3.34	-0.27	0.60	0.06	aTSS of 00721
1160259		+	2.58	1.80	-0.20	0.04	iTSS of 01059
1868809		+	3.54	0.06	0.00	2.06	aTSS of 01738
2481274		-	4.91	-0.27	0.00	0.57	aTSS of 02341
3198577		+	3.66	-1.12	-0.12	-0.09	aTSS of 03043
**3294538**		-	1.12	0.28	0.30	0.34	Long 5′UTR of 03142
3328034		-	2.35	0.51	1.96	2.69	aTSS of 03169
3579018		-	5.11	1.83	2.50	0.59	aTSS of 03906
3579126		-	4.69	1.36	2.07	0.68	aTSS of 03906
3579259		-	4.81	1.33	1.90	0.54	aTSS of 03906
3722203		-	5.26	0.69	0.00	1.04	aTSS of 03567
3946388		+	3.19	-1.25	3.63	-0.12	iTSS of R0092

Supplementary Table S6: Genes putatively transcribed from multiple TSSs and their corresponding alternative TSS positions. Previously known TSSs are shown in bold.

**Accession N°**	**Gene**	**Strand**	**Start codon position**	**TSS 1**	**TSS 2**	**TSS 3**
CCNA_00011	*dnaJ*	+	10223	10178	10092	10075
CCNA_00721	*groEL*	-	779585	779585	779608	779703
CCNA_01261		+	1388006	1387963	1387950	
CCNA_01341		+	1454414	1454394	1454375	1454280
CCNA_02039	*clpX*	-	2185128	**2185230**	**2185325**	
CCNA_02552	*clpS*	+	2699457	**2699418**	2699367	**2699283**
CCNA_02845	*uzcR*	-	2998187	**2998223**	**2998266**	
CCNA_02846		-	3000222	3000341	**3000364**	
CCNA_02860		-	3013060	**3013083**	3013098	
CCNA_03299		+	3472717	**3472677**	3472603	
CCNA_03362	*sigF*	-	3541812	**3541814**	3542003	
CCNA_03483		-	3646517	**3646553**	3646572	
CCNA_03706		+	3874686	3874440	3874475	**3874612**
CCNA_R0095		-	4950	5035	5053	5188

microorganisms

MDPI

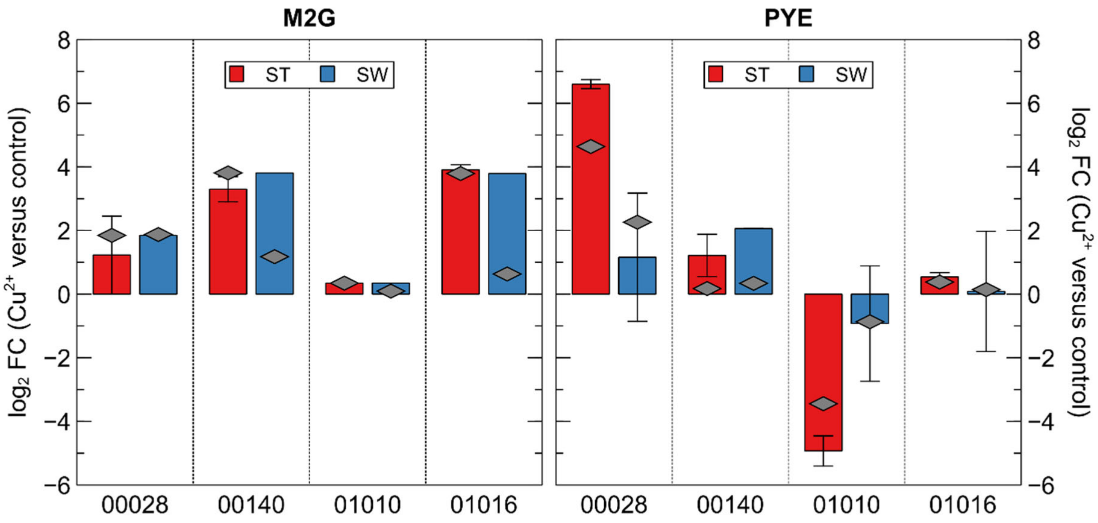

Supplementary Figure S1: Differential expression of 4 selected genes, in stalked cells (ST) and swarmer cells (SW) in 2 culture media (M2G and PYE). Bars represent log2 fold change values from qRT-PCR data, with diamonds representing log2 fold changes calculated with tagRNA-seq. CCNA accession numbers are shown at the bottom.
